# Factors Associated with Abnormal Mammogram Results Among Low-Income Uninsured Populations in Medically Underserved And Rural Texas Regions

**DOI:** 10.1089/whr.2024.0048

**Published:** 2024-09-06

**Authors:** Wen Hsin Chen, Arica Brandford, Rosaleen Bloom, Gang Han, Scott Horel, Marivel Sanchez, Anna Lichorad, Jane Bolin

**Affiliations:** ^1^ Department of Health Policy and Management, School of Public Health, Texas A&M University, College Station, Texas, USA.; ^2^ Houston Methodist Research Institute, Texas A&M University, Houston, Texas, USA.; ^3^ School of Nursing, Texas A&M University, College Station, Texas, USA.; ^4^ Department of Epidemiology and Biostatistics, School of Public Health, Texas A&M University, College Station, Texas, USA.; ^5^ School of Public Health, Texas A&M University, College Station, Texas, USA.; ^6^ School of Medicine, Texas A&M University, College Station, Texas, USA.

**Keywords:** breast cancer, mammography, screening, population health, community health

## Abstract

**Background:**

This study investigated the potential associations between neighborhood characteristics, rurality, ethnicity/race, and breast cancer screening outcomes in designated Health Professional Shortage Areas in Central Texas. Limited access to preventive medical care can impact screening rates and outcomes. Previous research on the effects of factors such as rurality, neighborhood socioeconomic status, and education level on cancer prevention behaviors has yielded inconsistent results.

**Materials and Methods:**

We analyzed data from a state-funded breast and cervical cancer screening programs for disadvantaged and medically underserved individuals. A mixed-effects logistic regression model was used to assess the impact of residency characteristics (rurality, educational attainment, unemployment, and poverty) on abnormal breast cancer screening outcomes, with individual level (age, ethnicity, race, and education) as control variables.

**Results:**

During the studied time, there were 1,139 women screened and 134 abnormal mammograms found. Residency characteristics were not significantly associated with abnormal mammography outcomes at 0.05. However, individual factors are strongly associated with abnormal screening results. Non-Hispanic or Latino white women had increased odds of abnormal clinical outcomes compared with Hispanic or Latino women (OR = 2.03, CI 1.25–3.28; *p* = 0.004). Additionally, women residing in counties with more than 30% of the population completing college had increased odds of abnormal mammogram outcomes compared with counties with less than 15% college attainment (OR = 2.89, CI 0.99–8.38; *p* = 0.051).

**Conclusions:**

This study found a significant correlation between area-level educational characteristics and abnormal mammography outcomes. Future research should explore the contextual risk factors influencing breast cancer occurrence and develop targeted interventions for this population.

## Introduction

One in eight women will be diagnosed with breast cancer during their lifetime. Breast cancer is the second leading cause of cancer-related death among women in the United States (U.S.).^1^ In 2023, it is estimated that 297,790 women will be diagnosed with invasive breast cancer, and approximately 43,700 women will die of breast cancer.^1^ The early detection of breast cancer is a critical determinant of survival. Approximately 99% of patients with a localized (nonmetastatic) breast cancer diagnosis survive for more than five years. However, the 5-year survival rate for late-stage diagnosis has decreased to 39%.^2,3^ Populations living in disadvantaged/underserved areas report lower screening uptake rates.^4–9^ Consequently, these residents often have disproportionately poor prognoses and high mortality rates.^10–13^

Disadvantaged or medically underserved counties and communities may be characterized by poverty, low educational attainment, rural location, and a high rate of uninsured individuals.^14,15^ Subsequent health disparities result from various factors, such as the adoption of state Medicaid expansion, residential segregation, and neighborhood resources.^16–20^ The multidimensional nature of residing in these areas often negatively impacts cancer prevention behaviors and results in delayed detection compared with individuals residing in more affluent areas.^21,22^ There is ample evidence indicating areas that are rural, impoverished, and have lower educational attainment exhibit disproportionately lower rates of cancer screening utilization and higher breast cancer mortality.^11–29^ Rural–urban disparities also exist, with rural women having a higher risk of being diagnosed with breast cancer at a later stage owing to noncompliance with screening, lack of follow-up for abnormal test results, and limited access to quality care.^30^ Women living in poverty in counties with current or persistent poverty also have higher rates of late-stage diagnosis and cancer mortality.^10,12,26,31^ Similar disparities were observed among individuals residing in areas with lower educational attainment.^32^

However, research findings regarding breast cancer disparities are inconsistent. While some studies suggest urban women have denser breasts,^33,34^ other studies highlight that BMI and exposure to traffic-related pollutants would increase breast density and lead to higher breast cancer incidence rates in urban areas.^35–36^ Moreover, research indicates that there is no significant difference in the likelihood of receiving breast cancer screening between individuals residing in higher- and lower-poverty counties.^37^ Furthermore, other studies indicate that women with higher levels of education or residing in areas with higher educational attainment may have a higher incidence of breast cancer.^38,39^

The reasons for the observed disparities in breast cancer are not yet fully understood. However, they may be related to multilevel residential characteristics, such as living in higher poverty, high unemployment, or low educational attainment in counties and/or rural areas.^4,5,10–14,23–32^ Thus, this study aimed to examine the relationship between community or social determinants of health (SDoH) status and the risk of abnormal mammography screening results (suspicious malignant findings or abnormalities) among low-income and uninsured women in 22 counties in Central Texas and to evaluate whether there is a correlation between community and individual risk factors with breast cancer screening outcomes.

To further explore how residential characteristics influence, more specifically, we exclude the impact of coverage, which is recognized as a strong predictor of access to preventive care in this study.^40^ The study aimed to provide empirical evidence of the relationship between suspicious mammography screening results and community-level risk factors in low-income, uninsured women in Texas.

## Materials and Methods

### Study population

The Texas A&M Cancer Screening, Training, Education, and Prevention Program (C-STEP) provides free breast cancer screening services to women who are uninsured and underinsured, have a household income ≤250% of the federal poverty level, and primarily reside in Health Professional Shortage Areas (HPSAs).^41^ From 2017 to 2023, C-STEP provided no-cost mammography services (*n* = 1,139) to women 40–74 years of age within a 21-county region of Texas. The mammography procedures adhered to the guidelines set by the U.S. Preventive Services Task Force (USPSTF).

### Measures and data sources

After Institutional Review Board approval, the C-STEP opened to recruitment from July 2017 and screening data were collected until June 2023. Recruiting events were held in 21 counties in Middle Texas, with 10 community health workers (CHW) and designated clinical sites involved. The CHW teams in the CPRIT program work closely with community partners to engage potential participants at various locations, including health fairs, food pantries, resource centers, and stores. Furthermore, clinical sites play a vital role in keeping CHWs updated with the latest screening information and facilitating a seamless referral system, linking CHW navigation with screening facilities. During the study period, a total of 5,211 individuals were directly approached by CHWs. Out of these, 3,765 individuals were excluded from the recruitment process for reasons such as not meeting the inclusion criteria or declining to participate, among other factors.

**FIG. 1.**
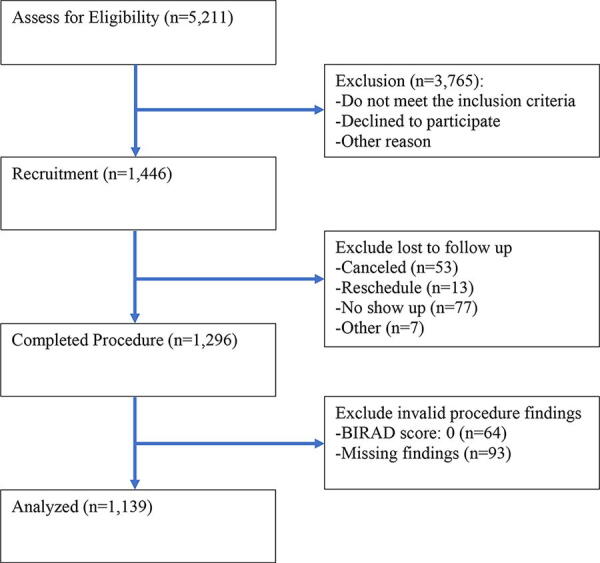
Flowchart illustrating the inclusion process for mammogram findings analyzed in a study on breast cancer screening among low-income, uninsured individuals in middle Texas from July 2017 to June 2023.

There are 1,446 participants who met the recruitment criteria and were referred by CHWs to designated clinical sites. We excluded those lost to follow-up such as individuals who canceled (*n* = 53), rescheduled (*n* = 13), not shown (*n* = 77), or other (*n* = 7). In total 1,296 participants completed procedures. Among those who completed procedures, 64 had invalid clinical findings (a BI-RADS score of 0, see description of scoring below) and 93 of the findings are missing. Subsequently, 1,139 participants’ findings are included in our analysis. See Figure 1 for the inclusion process.

The outcome variable is the mammogram results of participants. The mammogram result is based on the American College of Radiology Breast Imaging Reporting and Data System (BI-RADS).^42^ The BI-RADS score ranges from 1 to 6, with scores of 1–3 indicating normal and 4–6 indicating abnormal results. A BI-RADS score of 0 indicated an incomplete result that required follow-up; as described above, any sample with an invalid mammogram outcome was removed from the analysis. It is important to note some women underwent multiple breast cancer screenings in this program, and only the most recent valid mammogram outcomes were used in this study. Additional screening results were excluded because whether the procedures were routine screening, fixing incomplete procedures, or rechecking abnormal results was undeterminable.

The independent variables in this study are community-level risks, specifically focused on the rates of college attainment, unemployment, and poverty within the counties where the participants reside in Texas. Three key community-level indicators rely on data from the U.S. Department of Agriculture.^43^ Specifically, the poverty rate emerged as a significant metric, ascertained from the average proportion of adults residing below the poverty threshold within diverse counties from 2017 to 2021. The served counties exhibit an average poverty rate ranging from 8.9% to 23.9% and are subsequently categorized into three subgroups: less than 15%, between 15% and 20%, and exceeding 20%. The unemployment rate is determined by the average percentage of individuals unemployed between 2014 and 2022, dividing the metric into two groups: 5% or lower and higher than 5%. Educational attainment rate gauges the proportion of individuals completing collegiate-level education from 2017 to 2021. The variable was classified into five categories: below 15%, 15% to 19.9%, 20% to 24.9%, 25% to 30%, and surpassing 30%. Client residency rurality was categorized as a binary variable, distinguishing between urban and rural areas. This classification system is based on the Rural–Urban Continuum Code (RUCC) framework provided by the United States Census Bureau.^44^ RUCC codes 1–3 are designated for urban areas located within metro regions with populations of less than 250,000 people, between 250,000 and 1 million people, and more than 1 million people, respectively, categorizing them as having metropolitan status (RUCC = 1–3). By contrast, RUCC codes 4–9 signify rural areas. Nonmetropolitan status is determined by combining counties with an urban population ranging from 2,500 to 19,999 people, counties with an urban population of 20,000 people or more, and entirely rural counties or urban counties with populations lower than 2500 people (RUCC = 4–9).

Women 40–74 years of age were advised to participate in biennial breast cancer screening in the demographic cohort. We followed the recommendation for this study, which included only women within the USPSTF recommended age range. The mean age of the participants was 52 years. We classified age groups under 40–50, 50–60, and 60–74 years because most participants were between 40–60 years of age, and only a limited number were under 40 or above 74 years of age. Ethnicity and race are consolidated into a categorical variable: Latino or Hispanic white, Non-Latino or Hispanic white, black, and Other/Unknown. The education variable is stratified into four distinct categories: below a high school degree, high school degree attainment, completion of a college degree, and unknown.

### Statistical analyses

We initiated the analysis by calculating the proportion of women with individual- and community-level characteristics within our sample and the proportion displaying abnormal mammogram results. Pearson’s chi-squared test or Fisher’s exact test was used to test the association between patient characteristics and abnormal mammogram results. We used generalized linear mixed-effects models with Bernoulli distribution and logit links for the multivariable analysis. We presented the estimated odds ratios (ORs) and their corresponding 95% confidence intervals (CIs) for patient characteristics related to abnormal mammogram screening.

Specifically, we implemented a mixed-effects logistic regression model to include all woman-level characteristics (age, race/ethnicity/education) as fixed effects and counties as random effects. We also extended our analysis by introducing additional fixed effects at community levels, including urban residence and county level, such as poverty, unemployment, and college attainment rates. To ensure the robustness of our results, we performed two sensitivity analyses. In the second sensitivity analysis, we focused on a larger age subset of our sample. This step was undertaken because some women under 40 or above 74 years of age were identified as having a potentially higher risk of breast cancer. A two-sided *p* value of less than or equal to 0.05 indicates statistical significance in all analyses. Statistical analyses were conducted using Stata software version 17 (Stata Corp., 2021).

## Results

### Descriptive characteristics

Our study comprised a sample size of 1,139 women undergoing mammography screenings, with outcomes classified as either normal or abnormal. Consequently, there were 134 abnormal mammogram cases included in this study. These abnormal mammogram outcomes were systematically analyzed based on the intricate interplay of individual and community characteristics within distinct strata. (Table 1)

**Table 1.** Descriptive Characteristics of Women Receiving Abnormal Mammogram Outcome

Characteristics/mammogram results	Total number (*n* = 1,139)	Normal (*n* = 1,006) *N* (%)	Abnormal (*n* = 133) *N* (%)	*p* value^b^
Age at mammogram				0.22
40–49	474 (42%)	427 (42%)	47 (35%)	
50–59	433 (38%)	374 (37%)	59 (44%)	
≥60	232 (20%)	205 (21%)	27 (21%)	
Education				0.13
Below high school degree	421 (37%)	382 (38%)	39 (29%)	
Completed H.S. degree	360 (32%)	307 (31%)	53 (40%)	
Completed College	47 (4%)	42 (4%)	5 (4%)	
Declined	311 (27%)	275 (27%)	36 (27%)	
Race/Ethnicity				0.006
Hispanic or Latino white	632 (55%)	575 (57%)	57 (43%)	
Non-Hispanic or Latino white	290 (25%)	238 (24%)	52 (39%)	
Black	111 (10%)	100 (10%)	11 (8%)	
Other/Unknown	106 (10%)	93 (9%)	13 (10%)	
County poverty rate^a^				0.31
<15%	266 (23%)	233 (23%)	33 (25%)	
15–20%	498 (44%)	434 (43%)	64 (48%)	
>20%	375 (33%)	339 (34%)	36 (27%)	
County unemployment rate^a^				0.24
≤5%	653 (57%)	583 (58%)	70 (53%)	
>5%	486 (43%)	423 (42%)	63 (47%)	
County College attainment rate^a^				0.91
<15%	242 (21%)	216 (21%)	26 (20%)	
15–19.9%	356 (31%)	311 (31%)	45 (34%)	
20–24.9%	73 (6%)	63 (6%)	10 (7%)	
25–30%	87 (8%)	77 (8%)	10 (7%)	
>30%	381 (34%)	339 (34%)	42 (32%)	
Urban–Rural				0.22
Urban	630 (55%)	563 (56%)	67 (50%)	
Rural	509 (45%)	443 (44%)	66 (50%)	

^a^ The U.S. Department of Agriculture collected community-level data. The county poverty rate is the average poverty rate for each county in Texas from 2017 to 2021. The county unemployment rate is the average unemployment rate for each county in Texas from 2014 to 2022. The collegiate-level attainment rate is the average collegiate-level attainment rate for each county in Texas from 2017 to 2021.

^b^ Chi-square and Fisher’s exact tests were conducted to test the association between listing variables and abnormal mammogram results.

Hispanic or Latino white women constituted approximately 55% of our sample, while non-Hispanic or Latino white women accounted for approximately 25% of the participants. African American women represented roughly a tenth of the sample, with the remaining 10% categorized as belonging to other races or declining to provide race information (*p* = 0.006). Educational attainment revealed that 32% of the population held only a high school degree, while 37% did not complete high school. Conversely, 4% of women had at least a college degree, and 27% declined to disclose their educational level (*p* = 0.13).

Approximately 42% of the participants were 40–49 years of age, 38% were between 50 and 59, and 20% were 60–74 years of age (*p* = 0.22). The residential distribution demonstrates that 33% reside in counties with a poverty rate exceeding 30%, 44% in areas ranging from 15% to 20%, and 23% in counties where fewer than 15% of adults live under the federal poverty line. (*p* = 0.31). Urban residency was evenly distributed among our sample, with approximately 55% classified as urban and 45% classified as rural (*p* = 0.22). The distribution of women across counties based on the college attainment rate revealed percentages of 21%, 31%, 6%, 8%, and 34% for counties with <15%, 15–19.9%, 20–24.9%, 25–30%, and >30% college attainment rates, respectively (*p* = 0.91).

### Model results

Table 2 presents the outcomes of our mixed-model analysis, specifically focusing on the second model encompassing both patient- and community-level predictors. Regional disparities in the abnormal mammogram results were identified (Table 2). Our findings underscore the likelihood of encountering an abnormal mammography outcome progressively increases with higher college attainment levels within women’s residential areas. Specifically, women residing in counties where over 30% of the population hold a college degree exhibit moderately elevated odds of experiencing abnormal mammogram results compared with counties with less than 15% college attainment (OR 2.84, CI 0.99–8.38, *p* = 0.055). Our model indicates that rural women have no difference obtaining abnormal mammogram results than their urban counterparts. Conversely, the unemployment rate within a region, whether exceeding 5% or not, does not yield a statistically significant impact on predicting abnormal mammography (OR 0.84, CI 0.46–1.52, *p* = 0.565).

**Table 2.** Mixed Method Multilevel Generalized Linear Regression with Logit Link Log, Odds Ratios of Women Receiving Abnormal Mammogram Results

Variable	Model 1 (adjusting for individual characteristics)			Model 2 (adjusting for individual and community characteristics)		
	OR	95% CI	*p* value	OR	95% CI	*p* value
Age group						
40–49	Reference			Reference		
50–59	1.24	0.81–1.89	0.326	1.21	0.79–1.86	0.381
≥60	0 .96	0.57–1.62	0.871	0.93	0.55–1.59	0.799
Education						
Below high school degree	Reference			Reference		
Completed H.S. degree	1.31	0.81–2.12	0.279	1.31	0.81–2.13	0.271
Completed College	0.77	0.27–2.13	0.610	0.72	0.26–2.04	0.540
Declined	1.11	0.68–1.82	0.669	1.10	0.67–1.80	0.712
Race/Ethnicity						
Hispanic or Latino white	Reference			Reference		
Non-Hispanic or Latino white	2.03	1.28–3.23	0.003	2.03	1.25–3.28	0.004
Black	1.00	0.49–2.03	0.997	1.07	0.52–2.20	0.855
Other/Unknown	1.41	0.74–2.70	0.301	1.42	0.75–2.72	0.285
County poverty rate						
<15%				Reference		
15–20%				1.35	0.61–3.01	0.463
>20%				0.70	0.3–1.65	0.415
County unemployment rate						
≤5%				Reference		
>5%				0.84	0.46–1.52	0.565
County College attainment rate						
<15%				Reference		
15–19.9%				1.27	0.66–2.36	0.494
20–24.9%				1.98	0.68–5.80	0.213
25–30%				2.13	0.65–6.99	0.211
>30%				2.84	0.99–8.38	0.055
Urban–Rural						
Urban				Reference		
Rural				0.63	0.31–1.27	0.199

Furthermore, the impact of poverty levels within counties on abnormal mammography results shows nuanced outcomes. Residing in counties with more than one in five adults below the poverty line does not show statistically significant impact on abnormal outcomes relative to counties with a poverty rate of less than 15%. Similarly, women living in areas with a 15–20% poverty rate have a comparable likelihood of abnormal mammogram findings to those in areas with less than 15% poverty. Shifting the focus to individual characteristics, the analysis in Table 2 reveals compelling patterns. Non-Hispanic or Latino white women demonstrated heightened odds of experiencing abnormal mammography results compared with their Hispanic or Latino white counterparts (OR 2.03, CI 1.25–3.28, *p* = 0.004). Conversely, being non-Hispanic or Latino black women did not have a statistically significant difference of abnormal outcomes compared with Hispanic or Latino white population. The odds ratio for individuals under 50 years of age versus those 50–59 years of age was 1.21 (CI 0.79–1.86, *p* = 0.381), and the odds ratio for individuals under 50 years of age versus those 60 years of age and above was 0.93 (CI 0.55–1.59, *p* = 0.799). Moreover, educational attainment at the high school or college level did not yield statistically significant impacts on abnormal mammogram results.

### Sensitivity analysis

A sensitivity analysis with only residents living in HPSA areas yielded results consistent with our primary findings. To account for potential variations, we conducted a sensitivity analysis encompassing a broader sample that included women <40 years of age and >74 years. This age group was deliberately included, as it lies beyond the recommended age for women at average risk for breast cancer. Within this expanded sample, the age ranged from 32 to 88 years, with an average age of 52. Remarkably, this extended sensitivity analysis’s overall rate of abnormal mammograms mirrored that in the main analysis at 11.94%. The final model was meticulously replicated in the sensitivity and main analysis samples. The coefficients remained remarkably consistent across both sets of models, further confirming the robustness and reliability of our findings.

## Discussion

Exploring the association between community-level risks and abnormal mammography outcomes is important for designing cancer screening promotion strategies for underserved populations. Our study used multilevel data from the C-STEP program to explore potential community-level predictors of mammogram diagnosis among low-income, uninsured women between 40 and 74 years of age living in HPSA areas in Texas. Our multilevel analysis found that approximately 11.6% of women who underwent mammography screening in this study had abnormal mammogram outcomes. The odds of abnormal findings were not significantly associated with community and certain individual risk factors. Hence, these factors can only explain certain variations observed across our program’s counties.

Previous research has investigated demographic elements, such as age, race/ethnicity, education, and residential attributes with breast cancer incidence, but not necessarily abnormal outcomes.^45^ Our study revealed that women 50–59 years of age exhibited slightly elevated odds of abnormal mammogram outcomes. In contrast, those 60 years of age and above showed marginally lower odds of such outcomes, although these distinctions lacked statistical significance. By contrast, the National Cancer Institute notes an escalating probability of breast cancer diagnosis with advancing age. The risk affected 1 in 42 American women in their forties, rising to 2.4%, 3.54%, and 4.09% for those in their 50s, 60s, and 70s, respectively.^46^ Discrepancies may arise from differences in age levels or the populations under study.

Our analysis showed non-Hispanic white individuals had twice the odds of experiencing abnormal outcomes compared with Hispanic white individuals, with statistical significance at the 5% level (*p* = 0.05). However, the odds of abnormal outcomes for African Americans and other/unknown racial groups were comparable to those for Hispanic white individuals. A recent breast cancer incidence report from the Susan G. Komen Foundation shows that non-Hispanic white individuals have a 1.14 times greater lifetime risk of breast cancer than Hispanic white individuals.^47^ Although our findings suggest that the NH population has a greater risk of breast cancer occurrence, this does not mean that promoting breast cancer screening should decrease emphasis on Hispanic individuals since Hispanic women are more likely to be diagnosed with breast cancer at an advanced stage.^48^ The occurrence disparities of breast cancer may be influenced by various factors such as genetics, cultural practices, health behaviors, and environmental factors. Within the Hispanic population, there is a notable inheritance of genetic factors that offer protection against breast cancer, resulting in lower incidence rates among certain Hispanic and Latino communities.^49^ This genetic advantage is often attributed to a combination of European and Native American genotypes. However, the prevalence of Native American genotypes and inherited BRCA mutations within this population can influence not only the incidence of breast cancer but also the estrogen receptor (ER) and progesterone receptor (PR) status, as well as the histology of the tumor. Despite the lower incidence, breast cancers among Hispanic populations often exhibit more aggressive progression and lower responsiveness to cancer therapies.^50^

In addition to genetic factors, Hispanics are more susceptible to obesity compared with non-Hispanic whites, which can exacerbate metabolic disturbances, complicate cancer treatment, lead to severe complications, and adversely affect quality of life.^51–53^ Furthermore, Latino populations often adhere to diets high in carbohydrates, have limited access to healthy foods due to economic constraints, and experience lower food quality due to acculturation. Research suggests that poorer health behaviors and extreme obesity are associated with the duration of immigration to the United States.^54–55^ The process of acculturation among Latino populations significantly influences dietary habits, obesity rates, and consequently, increases the risk of breast cancer.^51–55^ In addition, existing evidence has linked individual higher education levels with an increased risk of breast cancer.^38,56^

However, our study found no statistically significant differences in mammography results based on education levels. Among the women recruited for our study, the prevailing issue was limited access to care owing to financial or geographical constraints. Such factors could influence outcomes, with low income and lack of insurance acting as mediating variables in our model.^57^ Our research demonstrated a moderate yet significant correlation between the college attainment rate in patients’ residential areas and the odds of experiencing abnormal mammogram outcomes. Specifically, individuals residing in regions where 30% of the adults hold college degrees were 2.84 times more prone to abnormal outcomes than those where less than 15% of the population achieved such degrees. In comparison to those residing in areas with college attainment rates below 15%, individuals living in areas with college attainment rates of 15–19.9%, 20–24.9%, and 25–30% demonstrated odds ratios of 1.25, 1.98, and 2.13, respectively, for experiencing abnormal mammogram outcomes. This progressive rise aligns with increasing levels of educational attainment in geographic regions. The underlying factor behind this phenomenon could be that women with higher educational attainment might encounter higher exposure to carcinogens than those with lower educational levels. Factors contributing to this exposure could include alcohol consumption, hormone therapy usage, or residence/working in areas with higher air pollution levels, often indicative of greater job opportunities and heavier traffic.^58,59^

Previous research indicates that women in persistently disadvantaged or poverty-stricken areas experience higher breast cancer occurrence and poorer outcomes or prognosis.^12,59,60^ Financially disadvantaged individuals or those in health care resource-limited regions tend to lack screening knowledge and health care provider recommendations, have lower trust in medical care, and face substantial barriers such as transportation and insurance.^61,62^ This further results in poorer knowledge of cancer prevention behaviors and compliance with recommended breast cancer screenings. While our study did not identify any significant impact associated with higher poverty rates, county-level unemployment indices, or rurality on breast cancer screening outcomes, it should be noted that neighborhood characteristics may still play a crucial role in influencing cancer occurrence. As this funding was provided to target low-income populations, the absence of a higher-income population in the model may have impeded the association between residency characteristics and screening outcomes in this model.

### Limitations

First, our participant pool had undergone a form of “preselection,” as individuals were chosen based on specific socioeconomic criteria. These criteria encompassed a household income of ≤250% of the federal poverty level, alongside being uninsured, to qualify for recruitment into the program. Consequently, our sample exhibits homogeneity, primarily characterized by low socioeconomic status and residence in the HPSA regions. Furthermore, it remains unclear whether participants adhered to the breast screening guidelines as their previous screening and abnormal mammogram follow-up results at clinical sites. Uninsured women commonly encounter prolonged intervals before initiating follow-up procedures following an abnormal mammogram result.^63^ This delay in diagnostic mammogram results correlates with a higher proportion of diagnoses made at advanced stages.^64,65^ These factors could potentially introduce a confounding effect on the genuine influence of residency characteristics on breast cancer screening outcomes owing to their potential mediating role.^44^ Our program also provides free screening services to participants rather than considering the affordability of screening in the real world. The financial aspect of accessing breast cancer screening services may consequently impact their ability to access care, influencing screening outcomes. This consideration underscores the potential interplay between affordability, access to care, and the resultant screening outcomes. This sample characteristic may limit the generalizability of our study to other populations. Due to the absence of clinical outcomes, such as cancer detection, as an outcome variable, this study may face limitations in assessing the clinical significance of abnormal mammogram results.

In this study, we do not account for all variables that may affect the relationship between mammogram results and residency characteristics, such as adiposity and weight. Research indicates that obesity significantly increases false-positive mammography results compared with women with a BMI less than 25.^66^ Higher BMI is positively associated with breast density, which is a strong risk factor for breast cancer and is related to abnormal mammogram results.^67^ Obesity is not only associated with mammogram outcomes but is also influenced by geographic characteristics. Evidence shows that low socioeconomic communities with little access to nutritious foods, more common in high-density black and Hispanic populations, are associated with higher prevalence of obesity.^68,69^ The quality of health care environment also impacts the prevalence of obesity.^70^ Obesity acts as a mediator, influenced by residency, which further affects mammography results. Thus, obesity may impact the estimated effect of geographic characteristics.

In this research, the sensitivity analysis encompassing female participants below <40 years and those above >74 years yielded outcomes consistent with the primary analysis conducted on the constrained study cohort. Furthermore, the additional sensitivity analysis, which excluded women residing in nondesignated HPSA regions, demonstrated findings akin to the principal analysis conducted on the entire study population. This approach enabled in-depth exploration of the potential impact of HPSAs and age on abnormal mammogram outcomes.

## Conclusion

This study uncovered disparities in breast cancer screening outcomes at individual and community levels. Notably, community-level educational attainment rates continued to impact abnormal mammography screening rates, even after adjusting for individual characteristics and specific recruiting counties. Further investigation is warranted to delve into the intricacies of community-level educational attainment and its influence on breast cancer outcomes. Subsequent research should incorporate a broader range of individual-level factors using multilevel approaches to identify risk factors precisely. Such an approach would facilitate the development of targeted strategies to promote cancer screening in vulnerable populations.

## Abbreviations Used

BI-RADS: Breast Imaging Reporting and Data System
CHW: Community Health Workers
C-STEP: Cancer Screening, Training, Education, and Prevention Program
ER: Estrogen Receptor
HPSAs: Health Professional Shortage Areas
ORs: Odds Ratios
PR: Progesterone Receptor
RUCC: Rural-Urban Continuum Code
SDoH: Social determinants of Health
U.S.: United States
USPSTF: U.S. Preventive Services Task Force
